# Comparative Effects of Resistance Training Modalities on Mental Health and Quality of Life in Individuals with Spinal Cord Injury

**DOI:** 10.3390/sports13020060

**Published:** 2025-02-18

**Authors:** Lucas Vieira Santos, Karla Raphaela da Silva Ramos Freitas, Eveline Torres Pereira, Luciano Bernardes Leite, Pedro Forte, Claudia Eliza Patrocínio de Oliveira, Osvaldo Costa Moreira

**Affiliations:** 1 Department of Physical Education, Federal University of Viçosa, Viçosa 36570-900, Brazil; lucas.vieira@ufv.br (L.V.S.); freitas.karlaraphaela@gmail.com (K.R.d.S.R.F.); etorres@ufv.br (E.T.P.); luciano.leite@ufv.br (L.B.L.); cpatrocinio@ufv.br (C.E.P.d.O.); 2 Department of Sports, Instituto Politécnico de Bragança, 5300-253 Bragança, Portugal; 3 Department of Sports, Higher Institute of Educational Sciences of the Douro, 4560-708 Penafiel, Portugal; 4 CI-ISCE, Instituto Superior de Ciências Educativas do Douro (ISCE Douro), 4560-547 Penafiel, Portugal; 5 Research Center for Active Living and Wellbeing (LiveWell), Instituto Politécnico de Bragança, 5300-253 Bragança, Portugal; 6 Institute of Biological and Health Sciences, Federal University of Viçosa—Florestal Campus, Florestal 35690-000, Brazil; osvaldo.moreira@ufv.br

**Keywords:** functional capacity, paraplegia, eccentric resistance training, power training, quality of life

## Abstract

Background: Anxiety and depression are prevalent after spinal cord injury, impairing social participation and quality of life. Objective: This study aimed to investigate the effects of traditional resistance training (TRT), flywheel resistance training (FWRT), and high-velocity resistance training (HVRT) on the mental health and quality of life in individuals with spinal cord injury. Methods: Thirty-two participants were divided into TRT (n = 12), FWRT (n = 8), and HVRT (n = 12) groups, undergoing 8 weeks of upper-limb training twice weekly under super-vision. Training intensity and volume were progressively increased. The Hospital Anxiety and Depression Scale and SF-36 Questionnaire were used to assess outcomes. Results: Both the TRT and FWRT groups showed a reduction in HADS-D scores post-intervention (*p* < 0.001). The TRT group also presented a significant reduction in HADS-A scores post-intervention (*p* = 0.003). Concerning quality of life, after training, TRT showed improvements in social functioning (*p* = 0.013), FWRT improved scores in physical functioning (*p* = 0.002), bodily pain (*p* = 0.002), vitality (*p* = 0.046), and role emotional (*p* < 0.001), while HVRT enhanced role physical (*p* < 0.001), social functioning (*p* = 0.013), and role emotional (*p* < 0.001). Conclusion: Overall, TRT was the most effective in reducing anxiety and depression and enhancing quality of life, while FWRT showed notable gains in physical and functional capacity. HVRT demonstrated improvements primarily in role physical but was less effective in other domains.

## 1. Introduction

Spinal cord injury profoundly affects multiple aspects of health, directly impacting the quality of life of those affected [1,2,3]. Spinal cord injury is associated with a range of acute and chronic impairments, including pain, motor dysfunction, sensory deficits, muscle dystonia, fatigue, atrophy, and alterations in muscle fiber composition. These physical changes often lead to reduced strength, deteriorated body composition, and gradual declines in both functionality and independence, highlighting the extensive long-term challenges faced by individuals with spinal cord injury [4,5,6]. In a recent study, Lee et al. [7] found that functionality losses were associated with psychological issues like anxiety, depression, sleep disorders, and suicide risk. Functional deficits also influence community participation and employment, which also might lead to mental health disorders [8,9,10].

Evidence suggests that, compared with able-bodied individuals, people with spinal cord injury are more likely to experience anxiety and depression. Approximately 22% of people with spinal cord injury are estimated to experience anxiety, while depression affects around 30% of this population [10]. Furthermore, the research shows that anxiety and depression can affect both paraplegic and tetraplegic individuals similarly, with injury severity or physical limitations not being the primary factors in the development of psychological disorders after spinal cord injury [11,12].

Physical exercise plays a valuable role in recovery strategies and treatments aimed at improving functionality and independence in daily activities, fostering autonomy, and preventing secondary complications. Resistance training stands out as an effective and reliable approach that not only enhances body composition and strength but also has positive effects on the mental health of individuals with spinal cord injury [4,13,14,15]. Additionally, active exercise induces both peripheral and central effects, promoting neuroplasticity, the release of brain-derived neurotrophic factors, pain modulation, and the normalization of GABA–glutamate neurotransmission, which are mechanisms associated with improvements in physical and mental health [16,17]. These effects highlight the potential of exercise to address both functional and psychological challenges in individuals with spinal cord injury.

Among resistance training methods, two are particularly beneficial for individuals with spinal cord injury: flywheel resistance training (FWRT) and high-velocity resistance training (HVRT). FWRT offers unique advantages by combining eccentric overload with concentric actions, leading to improved strength and power output with lower energy demands. This method also supports high-threshold motor unit recruitment and contributes to increases in muscle mass, fascicle length, and tendon strength [18,19,20]. This aligns with the findings of Stone et al. [21], who demonstrated that eccentric resistance training in individuals with incomplete spinal cord injuries, performed twice weekly over 12 weeks, was effective in improving both eccentric and isometric strength.

Equally, HVRT has shown significant improvements in functional capacity, muscle strength, and power, with these benefits achieved through low-to-moderate training loads—an important advantage for individuals with spinal cord injury-related limitations [22,23]. Recently, a study conducted by Rodrigues et al. [14] demonstrated that high-intensity resistance training with progressive volume, performed twice weekly over 12 weeks, can improve or maintain body composition, enhance upper limb muscle power, anaerobic power, and explosive strength. These improvements positively impact functional capacity, promoting greater autonomy and reflecting improvements in the mental state and quality of life for individuals with spinal cord injury. These results highlight the potential of FWRT and HVRT as effective and accessible training methods for individuals with spinal cord injury, targeting both strength and functionality with notable efficiency. However, the impacts of resistance training modalities may vary based on the severity of spinal cord injuries and individual specific factors.

Although the benefits of FWRT and HVRT are well documented compared with traditional resistance training (TRT) or other forms of RT, a knowledge gap remains regarding their specific effects on individuals with spinal cord injury. Therefore, this study aims to compare the effects of TRT, FWRT, and HVRT on mental health (anxiety and depression) and quality of life. We hypothesize that all three training modalities will lead to improvements in the mental health and quality of life in individuals with spinal cord injury.

## 2. Materials and Methods

### 2.1. Experimental Approach to the Problem

This experiment aimed to compare the effects of TRT, FWRT, and HVRT on mental health and quality of life outcomes in people with spinal cord injury. Given TRT’s established effectiveness in this population, it was used as a control group to encourage participant engagement and facilitate attendance during initial and final evaluations. This approach also addressed the unique mobility and adherence challenges faced by the spinal cord injury population. Training protocols followed principles designed to sustain intensity and gradually increase training volume, optimizing the training load throughout this study. Participants were fully briefed on this study’s procedures, with opportunities provided to address questions individually or in group settings.

### 2.2. Subjects

**Figure 1:**
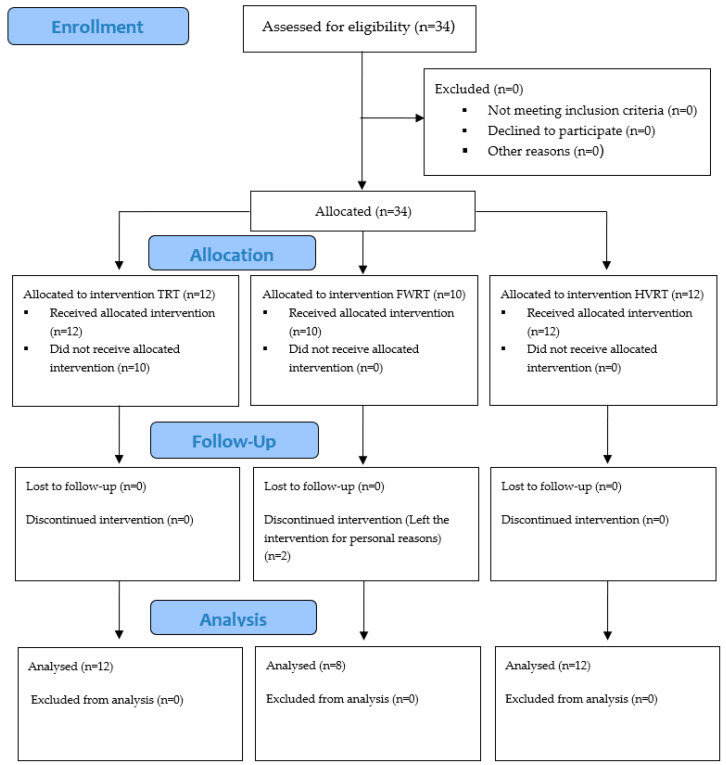
Study trial design. Overview of volunteer group allocation and dropout information. Flywheel resistance training (FWRT), high-velocity resistance training (HVRT), traditional resistance training (TRT).

The sample consisted of 34 individuals familiar with the university’s programs for people with disabilities, alongside additional participants recruited through a partnership with the city hall. Inclusion criteria required a clinically confirmed spinal cord injury, no upper limb musculoskeletal injuries within the past year, no prior RT experience, and a commitment to attend at least 80% of sessions without beginning other exercise programs. Participants were also asked to maintain their regular diets. Participants’ ages ranged from 28 to 65 years (49 ± 11.78), with time since injury averaging 22 ± 17.32 years, and an average weight of 64.62 ± 3.33 kg (Figure 1).

Throughout this study’s conception, potential inclusion and exclusion criteria and their possible impacts on the results were widely discussed. However, it is well known that individuals with spinal cord injury may develop various secondary effects, such as hypertension, anxiety, and depression. Additionally, these individuals face significant mobility challenges, which represent a major exclusion factor. For this reason, it was decided that only musculoskeletal injuries in the upper limbs would constitute grounds for volunteer exclusion.

Spinal cord injuries are highly unpredictable, as two similar injuries can lead to distinct sequelae, such as muscle rigidity or flaccidity. Considering these characteristics, the lead researcher responsible for the initial contact with the volunteers conducted an interview to better understand each individual’s potential and limitations. After all interviews were completed, it was determined that grouping participants based on injury level would be sufficient to minimize biases in the results.

During the initial contact between the volunteers and the researcher responsible for group allocation, a brief interview was conducted, during which the informed consent form was presented, and any questions regarding the assessments, training period, and other relevant details were clarified.

At this stage, the researcher collected documents confirming the spinal cord injury and discussed each volunteer’s limitations and potential abilities. All participants had complete injuries resulting from firearm wounds or automobile accidents. Furthermore, the researcher observed that the volunteers had traumatic spinal cord injuries at different levels, with lesion sites ranging from T4 to L1 vertebrae.

Given the diversity in participants’ physical conditions, randomization was not applied. Instead, the principal researcher assigned participants to groups to ensure homogeneity in terms of injury type and level, along with functional potential and limitations.

This grouping method avoided clustering similar injuries in one group, which might have skewed the results. The researcher responsible for group assignments did not participate in data collection or training sessions, contributing only to statistical analysis and interpretation of results. This research was previously approved by the Ethics Committee of the Federal University of Viçosa under the number 5.418.335 in May of 2022 and performed during the second semester of 2023.

### 2.3. Procedures

#### 2.3.1. Training Protocols

**Figure 2:**
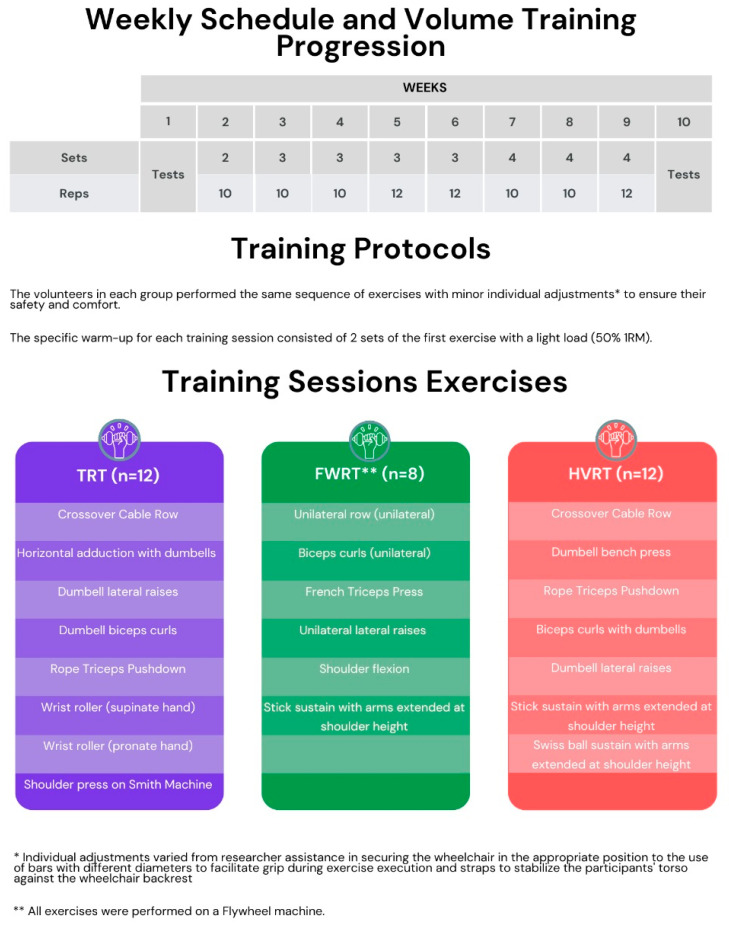
Description of the exercises performed by each group and weekly training volume throughout the intervention.

Thirty-four participants were divided into three groups: TRT (n = 12), FWRT (n = 10), and HVRT (n = 12). In the FWRT and HVRT groups, the focus was on executing the concentric phase of each movement as quickly as possible. FWRT participants followed the machine’s motion during the eccentric phase and braked in the last third to maximize eccentric overload benefits from the inertial flywheel machine (multi-leg isoinertial, Physical Solutions, São Paulo, Brazil). HVRT participants performed the eccentric phase in a controlled manner, not exceeding 2 s. TRT participants maintained a 2 s duration for both concentric and eccentric phases. The training period lasted eight weeks, with two sessions per week, progressively increasing in duration from 25 to 50 min from the first to the last week. Each session included two warm-up sets with a light load (50% of the training load). The training volume ranged from 2 sets of 8 repetitions to 4 sets of 12 (see Figure 2), and the training sessions focused on exercises for the functional upper body muscles and specific individual needs. Individual adjustments varied from researcher assistance in securing the wheelchair in the appropriate position to the use of bars with different diameters to facilitate grip during exercise execution and straps to stabilize the participant’s torso against the wheelchair backrest. Accordingly, all three groups performed exercises targeting the back, pectoralis major, deltoids, biceps, triceps, forearms, and core muscles. All participants performed the same exercises in the same sequence, with minor adjustments to enhance their safety and comfort. Training intensity was monitored using the OMNI-RES scale (1 to 10), with perceived exertion maintained between 7 and 9, and a 1 min rest was allowed between sets. All sessions and evaluations took place in the Department of Physical Education at the Federal University of Viçosa.

#### 2.3.2. Mental Health and Quality of Life Assessment

For the assessment of mental health and quality of life, two questionnaires were administered. The Hospital Anxiety and Depression Scale (HADS) was used specifically to measure symptoms of anxiety and depression, in its Brazilian validated version [24]. This scale includes 14 multiple-choice items divided into 2 subscales, 1 for anxiety and 1 for depression, each containing 7 items scored from 0 to 3. The total score for each subscale ranges from 0 to 21, with a cutoff score of ≥8 indicating possible anxiety or depression. Designed for use in non-psychiatric settings, HADS is a brief user-friendly tool, allowing patients to respond based on their experiences over the previous week. In addition to HADS, the SF-36 questionnaire was administered to evaluate quality of life across eight domains. This instrument is based on a thorough review of previous tools and is designed to capture changes in health, functional limitations, and social factors. Scores on SF-36 range from 0 to 100, with higher scores indicating a better quality of life [25]. Together, HADS and SF-36 provide a comprehensive assessment of mental health and quality of life, reflecting both emotional well-being and functional status.

### 2.4. Statistics

For the statistical analysis, descriptive statistics were initially applied to summarize the data. The Shapiro–Wilk test assessed data normality, and for variables that did not meet the normality assumption, a log transformation (base 10) was applied to normalize the data, allowing for the consistent use of parametric tests across all analyses. Box’s M test was used to evaluate the homogeneity of variances. Group comparisons at baseline were conducted with a one-way analysis of variance (ANOVA), followed by Bonferroni post hoc analysis. To examine intra- and intergroup differences over time, a two-way repeated measures ANOVA was used, incorporating two factors: time (pre- and post-intervention) and condition (comparing TRT, FWRT, and HVRT groups). A significance level of *p* < 0.05 was established for all tests. Analyses were performed using SPSS software, version 21.0.

## 3. Results

### 3.1. Mental Health

**Figure 3:**
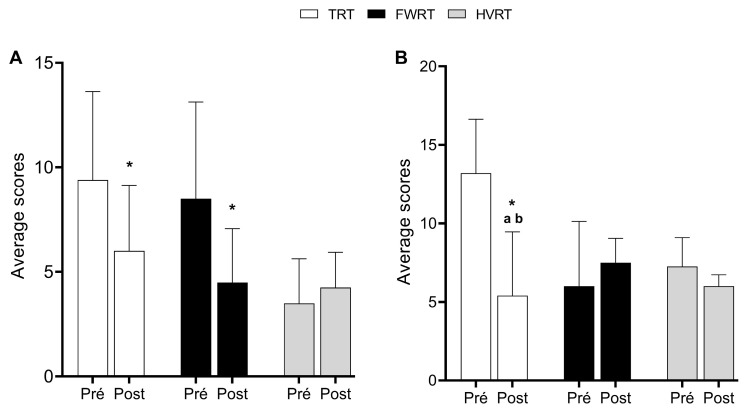
Hospital Anxiety and Depression Scale (HADS). (**A**) Depression (HADS-D). (**B**) Anxiety (HADS-A). Data are presented as means ± standard deviation. * *p*  <  0.05 vs. post intervention; ^a^ *p*  <  0.05 vs. FWRT; ^b^ *p*  <  0.05 vs. HVRT. Two-way repeated measures ANOVA followed by Bonferroni post hoc analysis.

The TRT and FWRT groups showed a reduction in HADS-D (Figure 3A) values at the post-intervention time point compared with the pre-intervention time point (*p* < 0.001; η^2^ = 0.776; β = 1.00). Specifically, the TRT group also exhibited a reduction in HADS-A (Figure 3B) values at the post-intervention time point compared with the pre-intervention time point (*p* = 0.003; η^2^ = 0.517; β = 0.931). Furthermore, in the comparison between groups at the post-intervention time point, it was observed that the TRT group had lower HADS-A values compared with the FWRT and HVRT groups (*p* < 0.001; η^2^ = 0.720; β = 0.995), indicating a moderate to large effect.

### 3.2. Quality of Life

**Figure 4:**
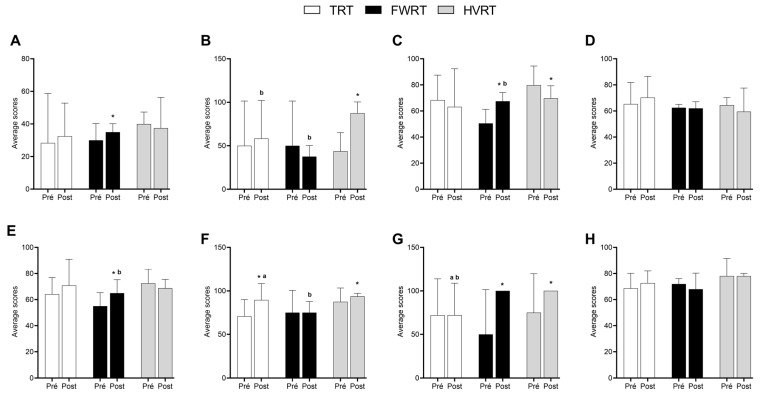
SF-36 health questionnaire. (**A**) Physical functioning; (**B**) role physical; (**C**) bodily pain; (**D**) general health; (**E**) vitality; (**F**) social functioning; (**G**) role emotional; (**H**) mental health. Data are presented as means ± standard deviation. * *p*  <  0.05 vs. post-intervention; ^a^ *p*  <  0.05 vs. FWRT; ^b^ *p*  <  0.05 vs. HVRT. Two-way repeated measures ANOVA followed by Bonferroni post hoc analysis.

Figure 2 presents quality of life data, with results indicating changes across various assessed domains. In the domain of physical functioning (Figure 4A), the FWRT group showed higher values after the intervention compared with the pre-intervention time point (*p* = 0.002; η^2^ = 0.500; β = 0.951), although no differences were observed between groups at the post-intervention time point (*p* = 0.306; η^2^ = 0.155; β = 0.234). For role physical (Figure 4B), the FWRT group showed higher values after the intervention (*p* < 0.001; η^2^ = 0.721; β = 1.00) and also compared with the TRT and FWRT groups (*p* < 0.001; η^2^ = 0.893; β = 1.00). In the bodily pain domain (Figure 4C), the FWRT group showed higher values, while the HVRT group showed lower values after the intervention (*p* = 0.002; η^2^ = 0.500; β = 0.951). Additionally, the FWRT group presented higher values at the post-intervention time point compared with the HVRT group (*p* = 0.001; η^2^ = 0.622; β = 0.977).

For general health (Figure 4D), no changes were observed over time or between groups (*p* = 0.958; η^2^ = 0.000; β = 0.050 and *p* = 0.248; η^2^ = 0.180; β = 0.273, respectively). In the vitality domain (Figure 4E), the FWRT group showed higher values after the intervention compared with the pre-intervention time point (*p* = 0.046; η^2^ = 0.240; β = 0.530) and lower values compared with the HVRT group (*p* = 0.047; η^2^ = 0.355; β = 0.598). For social functioning (Figure 4F), the TRT and HVRT groups showed higher values after the intervention (*p* = 0.013; η^2^ = 0.347; β = 0.752). Between groups, the FWRT group showed lower values compared with the TRT and HVRT groups (*p* = 0.008; η^2^ = 0.501; β = 0.861).

In the role emotional domain (Figure 4G), the FWRT and HVRT groups showed higher values after the intervention (*p* < 0.001; η^2^ = 0.582; β = 0.989). Additionally, the TRT group showed lower values at the post-intervention time point compared with the FWRT and HVRT groups (*p* = 0.004; η^2^ = 0.427; β = 0.877). Finally, in the mental health domain (Figure 4H), no changes were observed over time or between groups (*p* = 0.979; η^2^ = 0.000; β = 0.050 and *p* = 0.138; η^2^ = 0.124; β = 0.400, respectively).

No adverse effects related to the training were reported by the volunteers, regardless of the training group. This supports evidence indicating the safety and feasibility of the resistance training methods used in this study.

## 4. Discussion

This study investigated the effects of three RT protocols—TRT, FWRT, and HVRT—on the mental health and quality of life in people with spinal cord injury. The results demonstrated distinct benefits among the groups, indicating that different approaches may offer specific advantages in managing these outcomes, depending on the characteristics and needs of the individuals.

### 4.1. Mental Health

Initially, the TRT and FWRT groups showed a reduction in HADS-D values after the intervention, corroborating the literature that highlights the ability of RT interventions to mitigate symptoms of depression in populations with adverse health conditions [26,27,28]. Specifically, TRT also demonstrated a significant reduction in HADS-A values, results that are particularly relevant since depression and anxiety are often associated with a decreased quality of life and limitations in the social reintegration of people with spinal cord injury [29,30,31].

The intergroup analysis revealed that TRT was more effective in reducing HADS-A compared with the FWRT and HVRT groups, indicating a relevant effect. This advantage may be related to the simplicity and predictability of the TRT protocol, which creates a more comfortable and safer environment for practice, reducing stress levels associated with exercise. These findings reinforce the relevance of interventions that combine regular physical stimuli adjusted to individual capacities, especially in populations facing emotional and physical challenges due to the complexity of their conditions [32,33].

### 4.2. Quality of Life

Regarding quality of life, the results demonstrated specific benefits for each training protocol. TRT showed a significant increase in social functioning, highlighting its contribution to improving the social interaction of people with spinal cord injury. This type of training is recognized for fostering social interaction due to its structured environment and the encouragement of participation in group activities [34,35,36]. These same mechanisms may have contributed to the observed results in this population, promoting greater social engagement and support during the sessions.

Additionally, the FWRT group showed improvements in physical functioning, bodily pain, vitality, and role emotional, demonstrating its ability to promote enhancements in physical functionality, energy levels, and confidence in managing emotional limitations. Previous studies have already reported significant improvements in physical functionality and pain perception using this training method [14,37,38]. Therefore, this improvement in functional capacity may have contributed to the observed advancements in other domains by providing greater autonomy and facilitating the execution of daily tasks.

Furthermore, the HVRT group demonstrated improvements after the training period in the domains of role physical, role emotional, and social functioning, highlighting its positive impact on these aspects. These improvements may be related to the intense stimulus of the protocol, known for promoting gains in muscle functionality and quality of life by reducing perceived limitations in social and emotional interactions [39,40]. However, the significant increase in bodily pain may reflect the high physical effort required, emphasizing the importance of adjustments in intensity and progression to minimize discomfort and maximize benefits.

Building on these findings, although certain domains, such as general health, did not show significant changes, maintaining these aspects is already a positive outcome, considering the challenges faced by people with spinal cord injury. The varied impact of the protocols underscores the need for individualized interventions, integrating different training modalities to maximize benefits across multiple domains of quality of life.

Rivers et al. [41] identified a positive association between higher motor function (assessed by the functional independence measure (FIM)) and quality of life in the physical domain of SF-36V2, whereas a negative association was observed in the mental domain. While a lower FIM motor score reflects greater injury severity and poorer physical health, its correlation with higher self-reported mental health is less intuitive. Furthermore, Ditor et al. [42] demonstrated that adherence to exercise enhances quality of life, with significant benefits observed following a 9-month training intervention and an additional 3-month follow-up, including reductions in stress and pain, along with the promotion of an active lifestyle in individuals with spinal cord injury.

In the comparison between the groups, specific differences emerged in the evaluated domains. For instance, the HVRT group showed the best results in role physical and vitality, demonstrating its effectiveness in promoting functional and energetic gains. The FWRT group performed better in bodily pain, suggesting its potential for managing physical discomfort. In the domain of social functioning, TRT and HVRT outperformed FWRT, highlighting their ability to foster social interactions. In role emotional, FWRT and HVRT showed higher values compared with TRT, reinforcing their applicability in reducing emotional limitations. These findings suggest that each protocol can be tailored to specific needs, enabling personalized approaches.

### 4.3. Limitations and Strengths of This Study

This study presents some limitations stemming from the challenges of assembling a homogeneous group of people with spinal cord injury and related conditions, including factors such as varying times since injury, differences in trunk stability, and upper limb mobility levels. For this reason, participants were allocated to groups based on the assessment of an experienced researcher, aiming to balance the limitations and strengths present in a heterogeneous group. There was only one flywheel machine available in the laboratory where the FWRT was conducted, which limited interaction among volunteers during training sessions. This may have affected participants’ motivation throughout the training program.

Furthermore, the findings related to FWRT should not be associated with other types of eccentric training, as they represent distinct stimuli and should only be compared under equivalent conditions. It is also important to highlight the limitation in comparing TRT and FWRT, as there is no direct equivalence in execution speed and repetition duration. Therefore, being mindful of this, volunteers in the FWRT and HVRT groups were instructed and encouraged to perform the concentric phases at the highest possible speed, ensuring that all repetitions were executed at maximum or near-maximum intensity.

On the other hand, several strengths should be highlighted. Validated and widely recognized tools were chosen to assess mental health and quality of life. Furthermore, it is noteworthy that this is the first study to apply three different types of RT with similar training protocols in people with spinal cord injury, enabling a comparison between them. The present study also appears to be the first to use a flywheel machine and HVRT with this population. We hope this will encourage other researchers to further explore these methods and expand the range of training modalities available for individuals with spinal cord injury. Long-term studies spanning several months or years, along with follow-ups after training cessation, could be highly valuable for a deeper understanding of the findings presented in this study.

Despite its limitations, this study’s strengths help mitigate its impact and underscore its contributions. The heterogeneity in participant characteristics was addressed through careful group allocation, ensuring a balanced distribution of strengths and limitations. While limited flywheel equipment may have restricted interaction, this study’s pioneering use of flywheel resistance training in individuals with spinal cord injury paves the way for future research. Differences in execution speed and repetition duration between traditional resistance training and flywheel resistance training were managed by standardizing volume.

### 4.4. Practical Applications

The results of this study highlight the importance of integrating different resistance training modalities (TRT, FWRT, and HVRT) into rehabilitation programs for individuals with spinal cord injury, considering the rapid and effective potential of these interventions. Each modality demonstrated specific benefits that can be explored according to individual needs. TRT proved effective in reducing symptoms of anxiety and improving social interaction, making it an ideal option to promote greater social engagement in controlled environments. On the other hand, FWRT stood out for improving physical functionality and reducing pain perception, offering an efficient alternative for muscle recovery and the performance of daily activities. Meanwhile, HVRT presented significant benefits in vitality and overcoming emotional limitations, contributing to greater autonomy and confidence.

Furthermore, one of the main practical applications is the rapid positive impact provided by these modalities. Even with a short intervention period of just eight weeks, with two weekly sessions, significant improvements were observed. This effect can be enhanced with increased training frequency or prolonged practice periods, highlighting resistance training as an effective and accessible approach for this population. Thus, a personalized strategy, combining modalities and adapting the frequency and duration of training, can maximize the physical and psychological benefits for people with spinal cord injuries, promoting their rehabilitation and quality of life.

Beyond the physical improvements, the observed benefits extend to important psychopathological outcomes, such as reduced anxiety, enhanced vitality, and improvements in emotional resilience. These results demonstrate how resistance training contributes to better mood regulation, reduced emotional distress, and increased self-efficacy, creating a feedback loop where physical gains reinforce psychological well-being. The enhanced sense of control and competence may help individuals manage stress more effectively, supporting long-term adherence to rehabilitation programs. Improved social interaction, facilitated by reduced anxiety and increased physical functionality, can further promote reintegration into daily life and community activities, improving overall quality of life. This highlights resistance training as a multidimensional therapeutic tool that addresses both physical recovery and psychopathological well-being.

## 5. Conclusions

In our findings, we observed that not only TRT has positive effects but also FWRT and HVRT are training modalities that can be applied to people with spinal cord injury to improve their mental health and quality of life. As demonstrated in the results, each training type led to specific outcomes, which may indicate that varying training modalities could significantly benefit this population. This leads us to understand that individuals with spinal cord injury should engage in resistance training regardless of the specific type employed.

## Data Availability

The data that support the findings of this study are available from the corresponding author, (PF), upon reasonable request.

## Abbreviations

The following abbreviations are used in this manuscript:

FWRT	Flywheel Resistance Training
HVRT	High-Velocity Resistance Training
TRT	Traditional Resistance Training
HADS	Hospital Anxiety and Depression Scale
HADS-D	HADS Depression Score
HADS-A	HADS Anxiety Score
